# Modelling the impacts of COVID-19 on nurse workload and quality of care using process simulation

**DOI:** 10.1371/journal.pone.0275890

**Published:** 2022-10-13

**Authors:** Sadeem Munawar Qureshi, Sue Bookey-Bassett, Nancy Purdy, Michael A. Greig, Helen Kelly, W. Patrick Neumann

**Affiliations:** 1 Human Factors Engineering Lab, Toronto Metropolitan University (Formerly, Ryerson University), Toronto, Canada; 2 Daphne Cockwell School of Nursing, Toronto Metropolitan University (Formerly, Ryerson University), Toronto, Canada; 3 University Health Network, Toronto, Canada; Jonkoping University, SWEDEN

## Abstract

Higher acuity levels in COVID-19 patients and increased infection prevention and control routines have increased the work demands on nurses. To understand and quantify these changes, discrete event simulation (DES) was used to quantify the effects of varying the number of COVID-19 patient assignments on nurse workload and quality of care. Model testing was based on the usual nurse-patient ratio of 1:5 while varying the number of COVID-19 positive patients from 0 to 5. The model was validated by comparing outcomes to a step counter field study test with eight nurses. The DES model showed that nurse workload increased, and the quality of care deteriorated as nurses were assigned more COVID-19 positive patients. With five COVID-19 positive patients, the most demanding condition, the simulant-nurse donned and doffed personal protective equipment (PPE) 106 times a shift, totaling 6.1 hours. Direct care time was reduced to 3.4 hours (-64% change from baseline pre-pandemic case). In addition, nurses walked 10.5km (+46% increase from base pre-pandemic conditions) per shift while 75 care tasks (+242%), on average, were in the task queue. This contributed to 143 missed care tasks (+353% increase from base pre-pandemic conditions), equivalent to 9.6 hours (+311%) of missed care time and care task waiting time increased to 1.2 hours (+70%), in comparison to baseline (pre-pandemic) conditions. This process simulation approach may be used as potential decision support tools in the design and management of hospitals in-patient care settings, including pandemic planning scenarios.

## 1 Introduction

Excessive nurse workload leads to overtime, increased error rates, presenteeism, absenteeism, burnout, decaying worker morale, decreased performance and injuries including work-related musculoskeletal disorders (WMSD) [1–7]. Pre-pandemic overtime and absenteeism costs in Canada were $968 million dollars and $989 million dollars respectively in 2017, where 24,600 registered nurses (RNs) were absent weekly and upwards of 20 million overtime hours were needed [8]. Subsequently, 71% of nurses have faced burnout at least once in their career [9], and the healthcare sector ranked number one in lost-time injuries, including work-related musculoskeletal disorders (MSD) [10, 11]. The COVID-19 pandemic has brought immense pressure to an already overworked healthcare professional (HCP) workforce [12–14]. Due to the COVID-19 pandemic, HCPs now have a significantly increased workload, which includes increased infection prevention and control (IPAC) routines [15]. Unable to keep up with these work demands, 8.4% of nurses plan to retire, 7.2% plan to leave nursing in the next quarter [16] and 71% of RNs, surveyed in December 2020, report having experienced a “breaking point” [17]. The Registered Nurses’ Association of Ontario (RNAO) surveyed 1,910 nurses during January 29 to February 22, 2021, where 17.4% of the respondents reported leaving the nursing profession as a “very likely” outcome [16]. There is, however, a lack of measures/tools that quantify nurse workload impacts of the pandemic in contextually sensitive ways.

A further concern in the pandemic lies in the extra workload for HCPs associated with IPAC protocols. Pre-pandemic research suggests that HCP workload was already near a maximum [18]. The added routines of the pandemic, such as additional IPAC routines, and increased care duties due to increased patient acuity, further contributes to the overload and excess fatigue. While quantifying nurse workload is notoriously difficult due to the complexity of these systems, newer applications of nurse-focused computerized process simulation of nursing work have shown promise in this area [14, 18]. Several studies have measured HCP workload during the COVID-19 pandemic [19–22]. However, there are currently no computerized simulation methodologies approaches available to understand and quantify the unit specific, shift long indicators of HCP workload, and associated care quality implications on a task by task basis, under pandemic outbreak scenarios such as COVID-19. We used process simulation technologies to determine the impact of caring for COVID-19 patients on nurse workload and quality of care.

### 1.1 Computerized process simulation in healthcare

Discrete Event Simulation (DES) is an operations research approach [23], that can be used to assess and predict the behavior and efficiency of a proposed or an existing operations system [24, 25]. It is widely used in manufacturing and service industries [26, 27]. In healthcare, DES has mostly been used to model patient flow to improve patient throughput, scheduling of patient admissions, and minimize patient wait time, modelling operations in the perianesthesia units, emergency department, pharmacies, and for operating room scheduling [28–31]. These approaches have been limited to simulating the patient as “product” flow in a production system—but have not addressed task-level workload of the HCPs performing the work. Qureshi and colleagues [32] developed a nurse-focused approach to DES. They modelled from the perspective of a nurse by simulating the care delivery process of nurses and their interaction with the system design and organizational policies. Using this approach, they quantified the impact of nurse-patient ratio, patient acuity and geographical patient bed-assignment on nurse workload and quality of care indicators [18, 32, 33]. Qureshi and colleagues [14] adapted this modelling approach to a medical-surgical unit where it produced highly valid data when compared to actual care delivery data. This study extends this modelling approach by adapting this into the context of the COVID-19 pandemic.

The aim of this research, therefore, was to develop, validate and test a DES modelling approach that can quantify nurse workload and care quality parameters as the number of COVID-19 positive (C+) patients assigned to a nurse increases from 0 to 5 in a 5-patient assignment scenario for a specific hospital care unit. In doing this, the model will isolate the impacts of both the extra IPAC routines, and the increased patient severity associated with C+ patients on nurse workload and care quality outcomes.

## 2 Methods

### 2.1 Model creation

**Fig 1:**
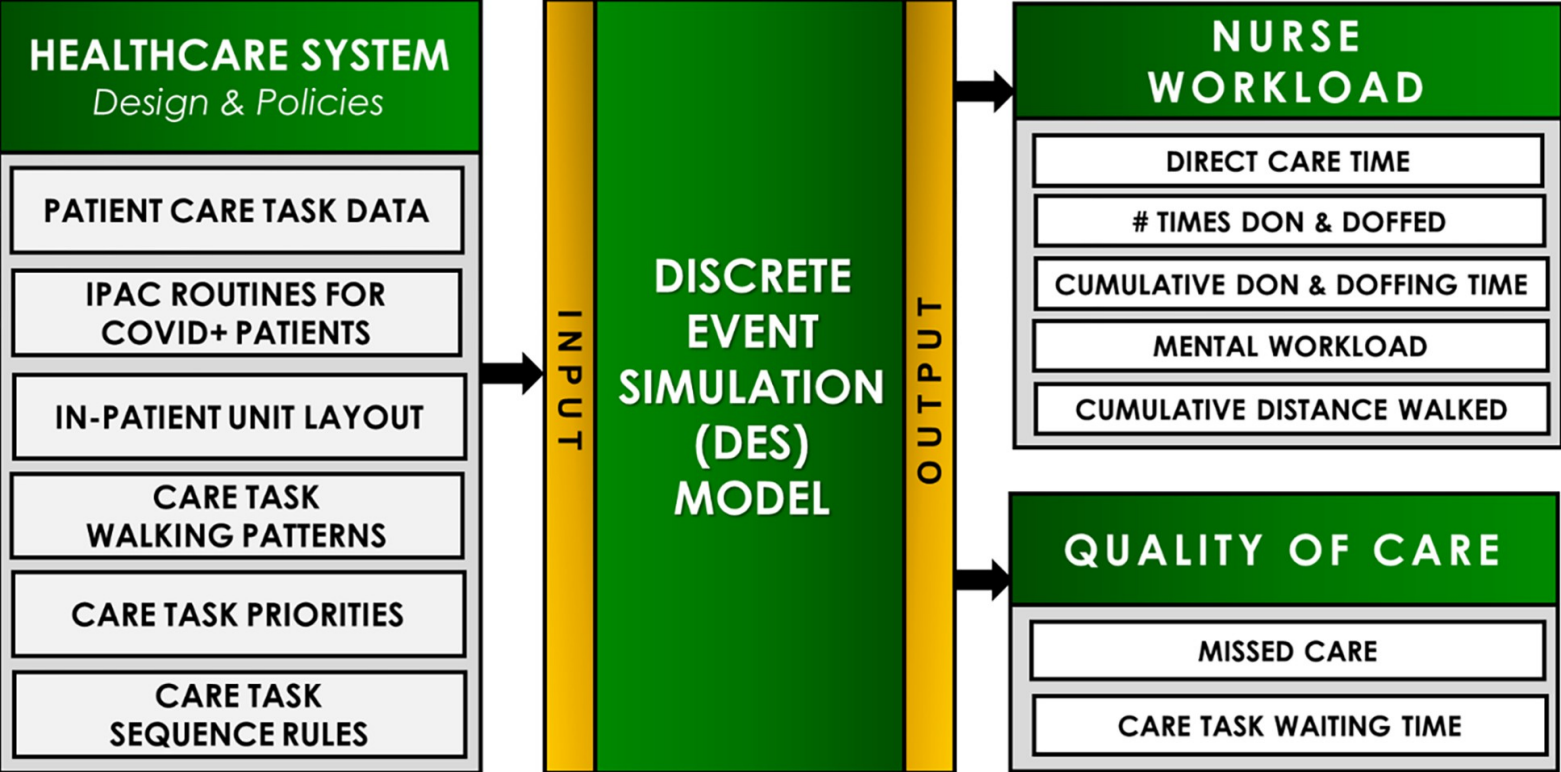
Overview of the model inputs and outputs. Where, Model Inputs are depicted as Healthcare System Design and Policies. Model Outputs include indicators of Nurse Workload and Quality of Care.

The DES model simulates the process of care delivery on a task-by-task basis. The model was created using the commercial version of Arena (Rockwell Automation). Modelling was conducted on a medical-surgical unit in a large urban teaching hospital that was upgraded to C+ status to accommodate the rapid influx of C+ patients. The unit was tasked with providing care to C+ patients during the second wave of the pandemic (October—December 2020). Research ethics approval was received from the healthcare institution and university research ethics boards. All participants were recruited using informed consent procedures. Fig 1 illustrates the inputs and outputs of the DES model. Inputs to the model include: 1) patient care task data, 2) IPAC routines for C+ patients, 3) in-patient unit layout, 4) programing logic that consists of a) care task walking patterns, b) care task priorities, c) care task sequence rules.

### 2.2 Model inputs

Model inputs (left side, Fig 1) were obtained from a number of data sources. These included institutional records, direct observations, the use of subject matter experts, and through a series of interviews and focus groups with nurses working in the hospital study unit. Specific details about modelling inputs are presented below.

#### 2.2.1 Patient care task data

*Patient care task data* defines the work to be performed by each nurse and is where the acuity differences between C- and C+ patients was established. This includes the care tasks delivered, their frequency and their duration. Anonymized patient care data, specifying the care tasks performed for each patient, were collected from an in-patient medical-surgical acute care unit in a large metropolitan area teaching hospital in Canada for a period of one year. The data collected were in the form of cost centre report data called Infor systems, more commonly known as the GRASP system [34]. The GRASP data consisted of standardized care task duration and task frequency [35].

Registered Nurses (RNs) in Canada must complete a four-year baccalaureate degree from a post-secondary university nursing program or a collaborative college-university nursing program [36]. RN education draws on areas of clinical practice, physical and biological sciences, critical thinking, ethics, research utilization, social and behavioral sciences and therapeutic relationships [37]. Nurses working in organizations that provide direct care in individual, family, group and community populations, must undergo additional training and shadow experienced nurses before being able to provide independent care in the unit. Occasionally, nurses must go through additional training with the introduction of new hospital policies (e.g. donning and doffing PPE protocol during the COVID-19 pandemic).

To understand how C+ patients have impacted the nurse’s ability to provide care, multiple focus groups were conducted (n = 5) with 12 experienced RNs with 2 to 27 years of experience. To ensure consistency, participants were selected from the same unit from where the patient care data and unit layout was collected. Nurses reported that some care tasks for C+ patients have increased frequency (up to 50%) while other tasks remain unchanged. Similarly, some task durations were noted to be longer for C+ patients. As an attempt to represent the most nurses in the modelling algorithms, the changes in acuity levels for C+ patients were determined by using mode value [38]. Participants with higher years of experience were given more weight in calculations. While it is possible to configure the model to individual nurse views and care delivery approaches to examine the impacts these differences have, the aim of this research was to develop a simulation approach to explore the impacts of the number of C+ patients on workload. Therefore, a single care delivery scenario representing a ‘typical’ nursing scenario for the unit, was used for all analyses. A summary of tasks and the differences between C+ and non-COVID-19 (C-) patients are presented in Table 1.

**Table 1:** Summary of the care task groups programmed into the DES model and their task duration, priority rank, and sensitivity to COVID-19 where C+ = COVID+ patients; C- = non-COVID patients. IPAC routines were necessitated by the pandemic and are specific for C+ patients only.

Task Category Name	*Non-COVID (C-) patients*		*COVID+ (C+) patients*		
	Time Duration (mm:ss) [Baseline]	Priority level (rank)	Priority level (rank)	Change in Time Duration [% increase due to COVID-19]	Change in Task Frequency [% increase due to COVID-19]
Activity	16:36	4	6	↑ (10%)	-
Assessment and Planning	02:30	1	1	↑ (20%)	↑ (20%)
Consultation	05:00	3	7	↑ (10%)	↑ (10%)
Elimination	09:42	4	3	-	-
Evaluation	03:00	3	10	↑ (20%)	↑ (20%)
Hygiene	1018	10	9	↑	-
Medication	15:00	2	2	↑ (25%)	↑ (25%)
Non-patient care	07:06	7	12	↑ (20%)	↑ (20%)
Nutrition	08:36	6	3	-	-
Other Direct Nursing Care	14:30	10	8*	-	-
Teaching and Emotional Support	20:54	8	2	↑ (30%)	-
Treatments	05:42	3	4	-	↑ (15%)
Vascular Access	06:36	2	5	-	-
Vital Signs	01:36	1	1	↑ (30%)	↑ (50%)
Admission	16:00	1	1	↑ (30%)	↑ (30%)
Discharge	10:42	16	11	-	-
IPAC routines	n/a	n/a	1	6:31 (mm:ss)	n/a

#### 2.2.2 IPAC routines for C+ patients

Hospital IPAC policy to protect against COVID-19 transmission required nurses to don and doff personal protective equipment (PPE) each time they entered or exited a C+ patient’s room. These posed additional tasks for nurses caring for C+ patients, compared to pre-pandemic work procedures and C- patients. The above-mentioned focus groups also revealed that RNs must wear a medical-grade safety mask throughout the shift. In addition, hospital policy dictates nurses must also wear safety glasses/safety shields, gown and gloves when attending to COVID-19 positive patients. The time duration required to don and doff the additional PPE was determined with a time and motion study observing 20 repetitions of donning and doffing with eight different nurse participants. These specific IPAC routines were necessitated by the pandemic and are specific for C+ patients only.

#### 2.2.3 In-patient unit layout

Unit layout, the physical architecture of the system, defines walking and transport distances in the unit. The unit’s physical dimensions were measured using a laser measuring instrument (Bosch GLM30 100 ft.). These dimensions were used to create a scaled layout drawing using Visio (Microsoft) and were then programmed into the DES model.

#### 2.2.4 Care delivery logic

The *care delivery logic* determines the sequence of tasks and associated movements of the simulant nurse to deliver the required care. The critical elements of this logic include a) care task walking patterns, b) care task priorities, and c) care task sequence rules.

- *Care Task walking patterns*–These entail the walking route of a simulant nurse while delivering a given care task. These require walking to the appropriate storage location if materials or medications are needed. Similarly, if spent or dirty materials must be disposed of after a care task, then this also requires walking to the disposal location on the unit. These required walking patterns were established in interviews with two subject matter experts (SMEs), RNs with 8 and 12 years of experience working in the study unit.
- *Care Task priorities–*These denote the relative importance or urgency of each care task, which was determined through a series of individual interviews and focus group discussions with 36 experienced RNs with 2 to 27 years of experience from the study unit. Nurses in each data-gathering session worked together to generate a priority ranking. A mode [38] priority level was calculated in cases when rankings differed across data collection events.
- *Care Task sequence rules–*This determines how the simulant nurse prioritizes and executes tasks in the DES model These were determined with the same group of nurses as for task priority. Nurses in all sessions unanimously agreed they deliver the highest priority task to the patient that is closest to their current location. However, when attending to C+ patients, nurses reported “bundling” as many care tasks as they can, so they would not have to don and doff PPE multiple times. These bundles of tasks were determined in the same data collection events with unit nurses, and the most common bundling strategy was used for the experimental analysis. It is noteworthy that, while it is possible to run the model to explore the impacts of different logics, priorities, or bundling strategies, the intent of this current study was to establish a consistent and reasonable baseline scenario that could be used for the comparative experiment.

These key inputs described above (left side of Fig 1) were then used to create a model of the unit in which a single simulant nurse delivers care to an assigned number of patients for a 12-hour shift. Below we describe model outputs, including nurse workload and quality of care indicators, that were obtained from the simulations, and then the outline of the model testing procedures.

### 2.3 Model outputs

Model outputs included indicators of workload and care quality. These are illustrated in Table 2.

**Table 2:** Indicators of nurse workload and quality of care quantified in this study.

Indicator Type	Name of Indicator	Description	Unit
**Nurse Workload**	*Cumulative Distance Walked*	The total distance a simulant-nurse walks during a 12-hour shift	km
	*Mental Workload*	The average stack of pending care tasks in a queue for a simulant-nurse. This represents the tasks that the nurse must “keep in mind” as they deliver care and represents an indicator of mental workload [10, 39, 40]	tasks
	*Direct Care Time*	The total time a nurse spent delivering care directly to patients, excluding non-direct care activities such as walking time and documentation	hours
	*Number of times Donned and Doffed*	The total number of occurrences in a 12-hour shift where a nurse had to don and doff PPE.	number
	*Cumulative Donning and Doffing Time*	The total time a nurse spent in donning and doffing PPE in a shift. These IPAC routines were necessitated by the pandemic and are specific for C+ patients	minutes
**Quality of Care**	*Missed care*	This is the number of care tasks that were not completed before the end of the simulated shift. The missed care tasks were also examined for the types of tasks missed	tasks
	*Care Task waiting time*	This indicator provides the average time a care task “waits’ in the queue before the simulant nurse can complete it. Lower task waiting times, with tasks performed as soon as possible, are associated with better care quality and improved patient outcomes [41]	hours

### 2.4 Model testing

Model testing consisted of three phases: a) model verification checks, b) model validation, and c) experimental tests of the impacts of the number of C+ patients in a standard five patient assignment.

#### 2.4.1 Model verification

Model verification consists of a series of checks aimed at confirming that the model operates as intended [42]. These were part of the model creation, and any flaws identified represent “bugs” in programming that were then corrected until all tests were satisfied. Verification tests were repeated after each model revision until all tests gave satisfactory results. The following model verification tests were done as recommended by Sargent [43]:

*Face validity–*The modelling results were shown to the subject matter experts (n = 8; nurses on the study unit) to determine if the DES model was producing realistic results [44]. They reported that the modelling outcomes were found to be producing results that resembled their work in real life.

*Extreme condition test–*The DES model was run on extreme conditions to check if the model would provide expected results. In this study, we tested two conditions: a simulant-nurse was assigned to 2 and 12 patients. As anticipated, the mental workload and care task waiting time increased extensively for 1:12 and decreased for 1:2 conditions compared to the 1:5 condition.

*Output relationship correctness test–*This technique explores the relationship of two dependent outputs. The DES model was run on the conditions 1:3, 1:4, 1:5 patients, all being C+. The number of times PPE is donned and doffed, and the cumulative donning and doffing time both saw equidistant increases.

#### 2.4.2 Model validation

To validate the model, a step counter test was used [14]. This test compared the modelling outcome “cumulative distance walked” with the real-world outcome “total distance walked”. Steps were measured in situ using a Fitbit^TM^ Alta Tracker with a step counter (c.f. Feehan and colleagues [45]) worn on the non-dominant hand during regular full-shift duties by 8 RNs (5 females, 3 males) with 2 to 15 years of experience in the modelled acute care unit. The Fitbit^TM^ Alta Tracker provides an accurate measurement of steps in adults in comparison to the distance walked [45]. Fitbit^TM^ devices use a 3-axis accelerometer to measure steps [46, 47]. The simulant-nurse was assigned to the same geographical bed location as the actual nurse to create consistency between real-world and modelling conditions. The number of steps walked by the actual nurse was converted into distance walked using the method of Zhang and colleagues [48]. Bartko’s [49] intraclass correlation coefficient (ICC) was used to estimate the similarity between modelling and real-world outcomes.

*Detailed information on the development of such modelling approaches*. A detailed explanation of the development of nurse-focused simulation models can be found at Qureshi [10] and Qureshi and colleagues [14].

#### 2.4.3 Experimental testing

**Fig 2:**
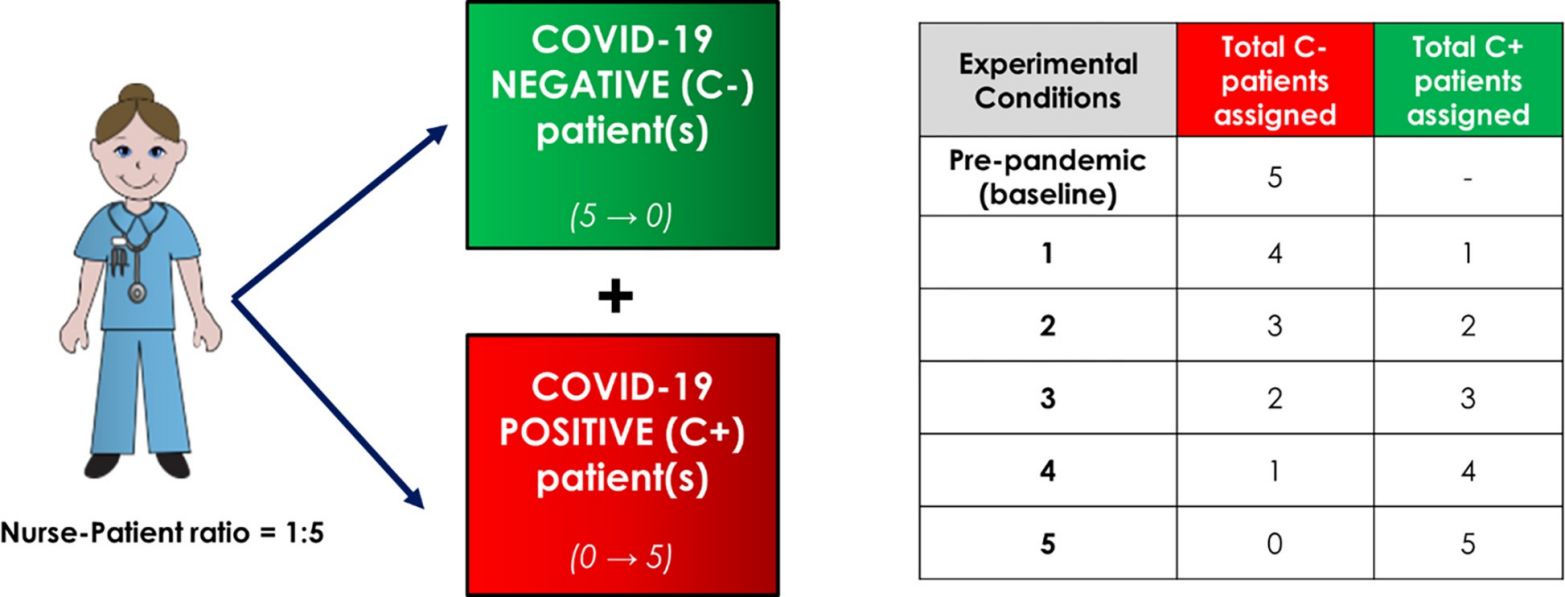
The experimental design conditions: A nurse assigned to the sum of C+ and C- negative patients where, zero C+ and five C- patients is the baseline (pre-pandemic) case. A nurse-patient ratio of 1:5 was kept for all conditions.

The nurse-patient ratio of 1:5 was kept in all conditions, a standard in most in-patient units [50]. In this study, experimental conditions span a broad range of C+ and C- patient bed assignments. For each condition, a nurse was assigned to the sum of C+ and C- patients (Fig 2) where the number of C+ and C- patients both varied from 0 (pre-pandemic, baseline case) to 5 (all C+ patients). The sum of C+ and C- patients for each condition totaled 5 patients.

Geographically, the simulant nurse was assigned to the typical bed assignment. In an interview with 19 RNs with 2 to 27 years of experience in the selected unit, they were shown a simplified blueprint drawing of the unit and were asked to draw the bed assignments for the past 10 shifts. Out of the 171 geographical bed assignments, the most common geographical shift assignment identified was selected. This assignment consisted of two beds near the nurse station, two beds far away from the nurse-station and one bed at an intermediate distance (between the far end and center of the unit). This represented a typical assignment on the unit and was kept constant for all tests.

The DES model represents “day” shifts, each consisting of 12-hours, with no breaks. Each condition was run for 365 shifts, calculated using the method of Banks and colleagues [23], to capture variability between shifts. The stochastic task arrival probabilities created some variation across runs. A warmup time of 92 shifts was used to estimate the optimal modelling state (c.f. Hoad and colleagues [51]).The average across the 273 post-warmup simulated days was calculated for all indicators with the differences in task arrival patterns for each simulated day providing the variability in each condition that can be seen in the results.

### 2.5 Ethical considerations

This study has been approved by the Research Ethics Board (REB) of Toronto Metropolitan University (formerly, Ryerson University) (REB approval # 2017–340), and field study site (reference # 20–5409). All participants provided written consent to participate in this study.

## 3 Results

### 3.1 Model validation test results

**Fig 3:**
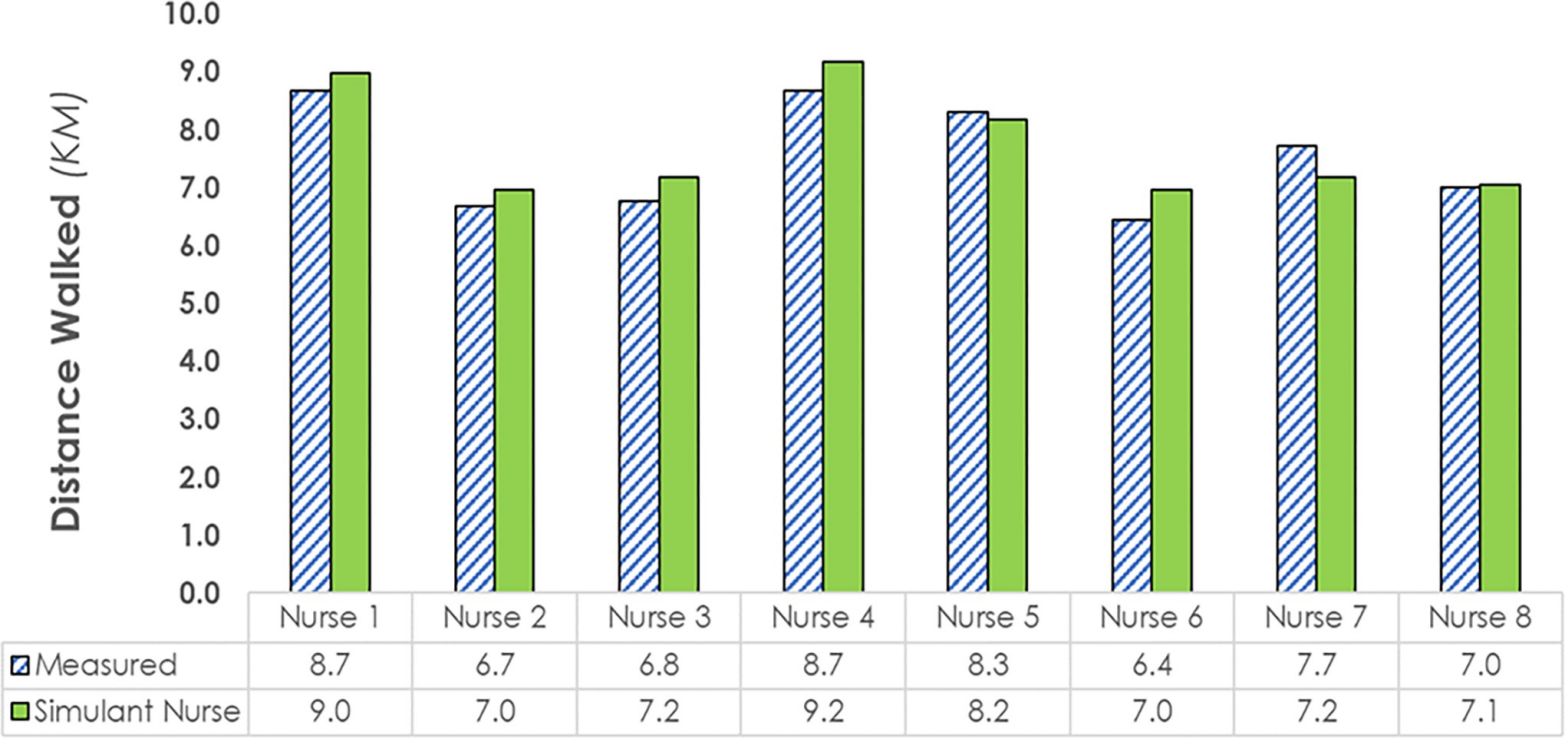
Distances walked by the simulant-nurse and the actual nurse (measured via step counter) during a 12-hour shift.

An overall ICC of 0.91 was observed for the comparison of actual nurse walking distances to those of the matched simulant-nurse. The simulant nurse walked an average of 7.7 km in a 12-hour shift (Range = 7.1 to 8.9; SD = 0.91), based on an average of 10041 steps (Range = 9166 to 11507; SD = 1174.6). In comparison, the actual nurses walked an average of 8.1km (Range = 6.44 to 8.68; SD = 1.68), based on 10567 steps (8367 to 11318; SD = 2179.2). Individual comparisons are presented in Fig 3.

### 3.2 Experimental results: Impact of COVID19 patient load

The DES successfully quantified the impact of COVID-19 patient assignments. The results are presented below.

#### 3.2.1 Nurse workload indicators

**Fig 4:**
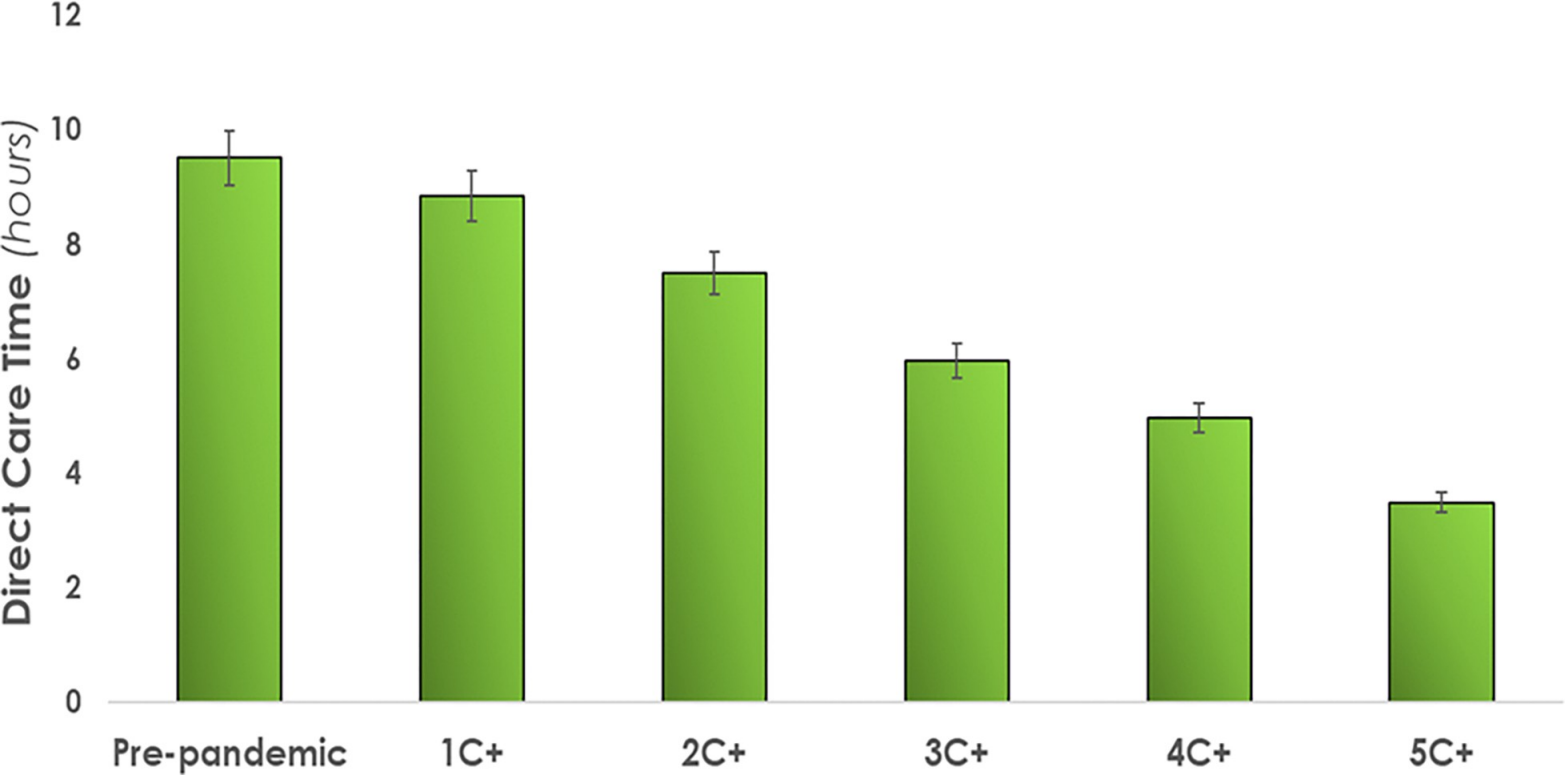
The impact of COVID-19 patient bed assignments on direct care time in relation to pre-pandemic conditions. Where, C+ = COVID+ patient. Error bars indicate standard deviations. Quantitative results are summarized in Table 3.

*Direct care time*. As illustrated in Fig 4, nurses spent a range of 4.1 hours to 8.8 hours delivering direct care when attending to C+ patients. Compared to the baseline pre-pandemic condition, a difference of -7% to -64% was observed across conditions.

**Fig 5:**
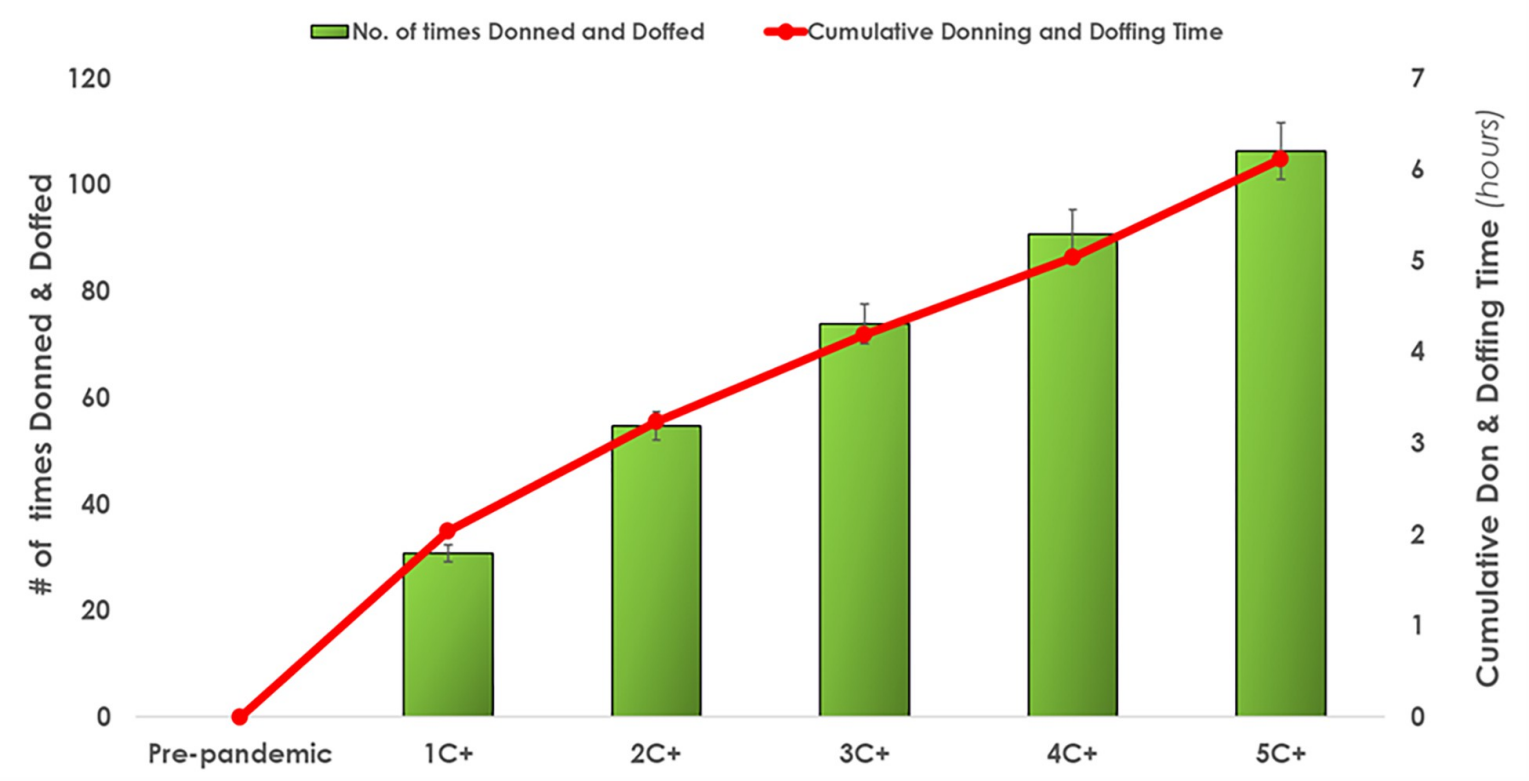
Average PPE donning and doffing frequency and cumulative time required in a single shift as COVID+ patients increased in a five-bed patient assignment. Error bars illustrate standard deviations. Table 3 provides a quantitative overview of the results.

*PPE donning and doffing*. Donning and doffing increased as C+ patients increased, ranging from 31 to 106 times in a 12-hour shift (donning and doffing = 1 count). Nurses spent a cumulative time of 2 hours (at 1C+)to 6.1 hours (at 5C+)donning and doffing. Fig 5 illustrates the relationship between ‘Number of times Donned and Doffed and ‘Cumulative Donning and Doffing Time’.

**Fig 6:**
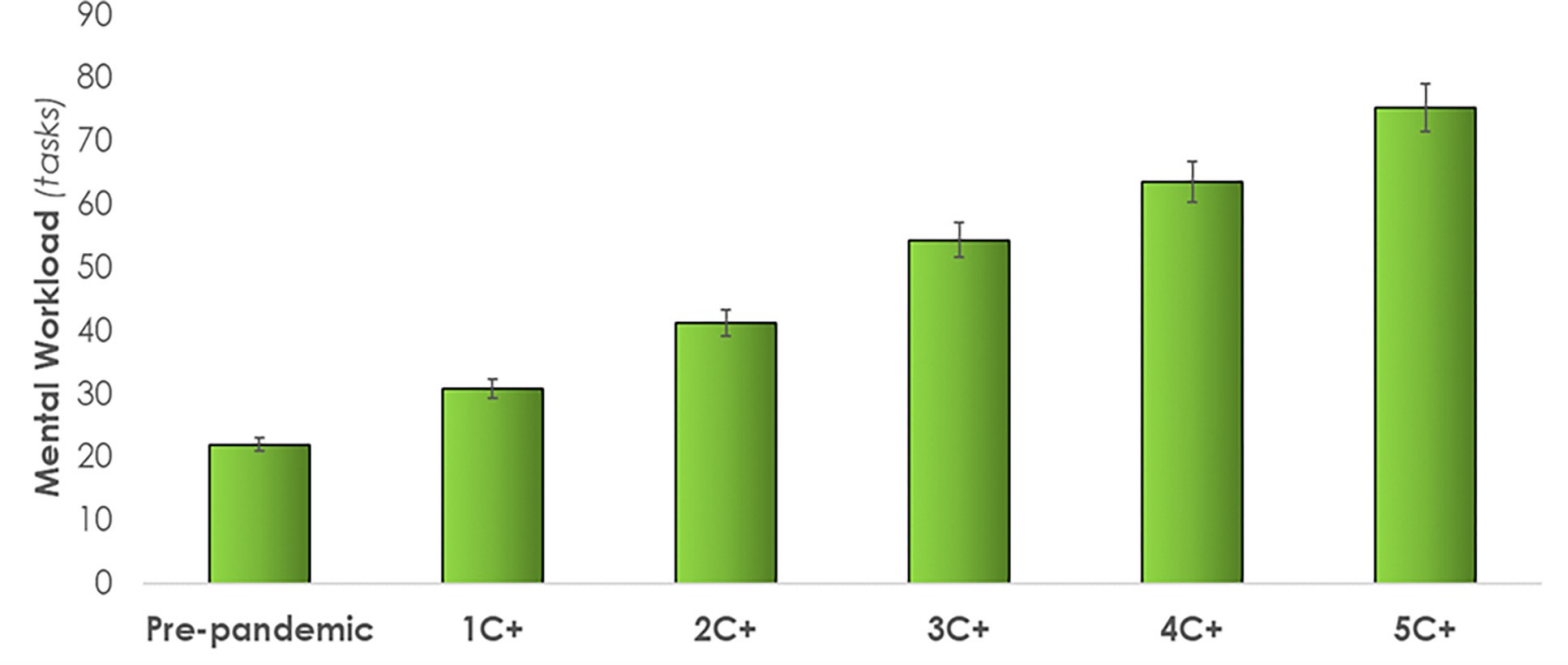
Mental workload increases with the number of COVID-19 positive (C+) patients assigned. Error bars indicate standard deviations. Table 3 provides a summary of the quantitative findings.

*Mental workload*. The average across shift number of care tasks “in queue”, a mental workload indicator, ranged from 22 at baseline, rising steadily to a shift average of up to 75 tasks waiting when assigned to 5 C+ patients (Fig 6). An increase of up to 242% from the baseline condition (pre-pandemic) was observed.

**Fig 7:**
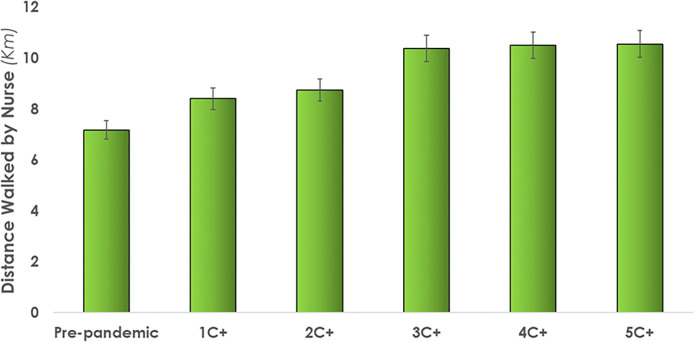
Average distance walked by nurses while attending to different numbers of c+ patients in their patient assignment. Error bars illustrate standard deviations. Table 3 provides a summary of the quantitative results.

*Cumulative distance walked*. When attending to C+ patients, a nurse walked a range of 8.4 km (1C+)to 10.55 km (5C+). A plateau effect was observed for conditions attending to three, four and five COVID-19 patients (Fig 7). An increase of up to 46% (seen in 5C+) was observed for the baseline pre-pandemic condition.

#### 3.2.2 Quality of care indicators

**Fig 8:**
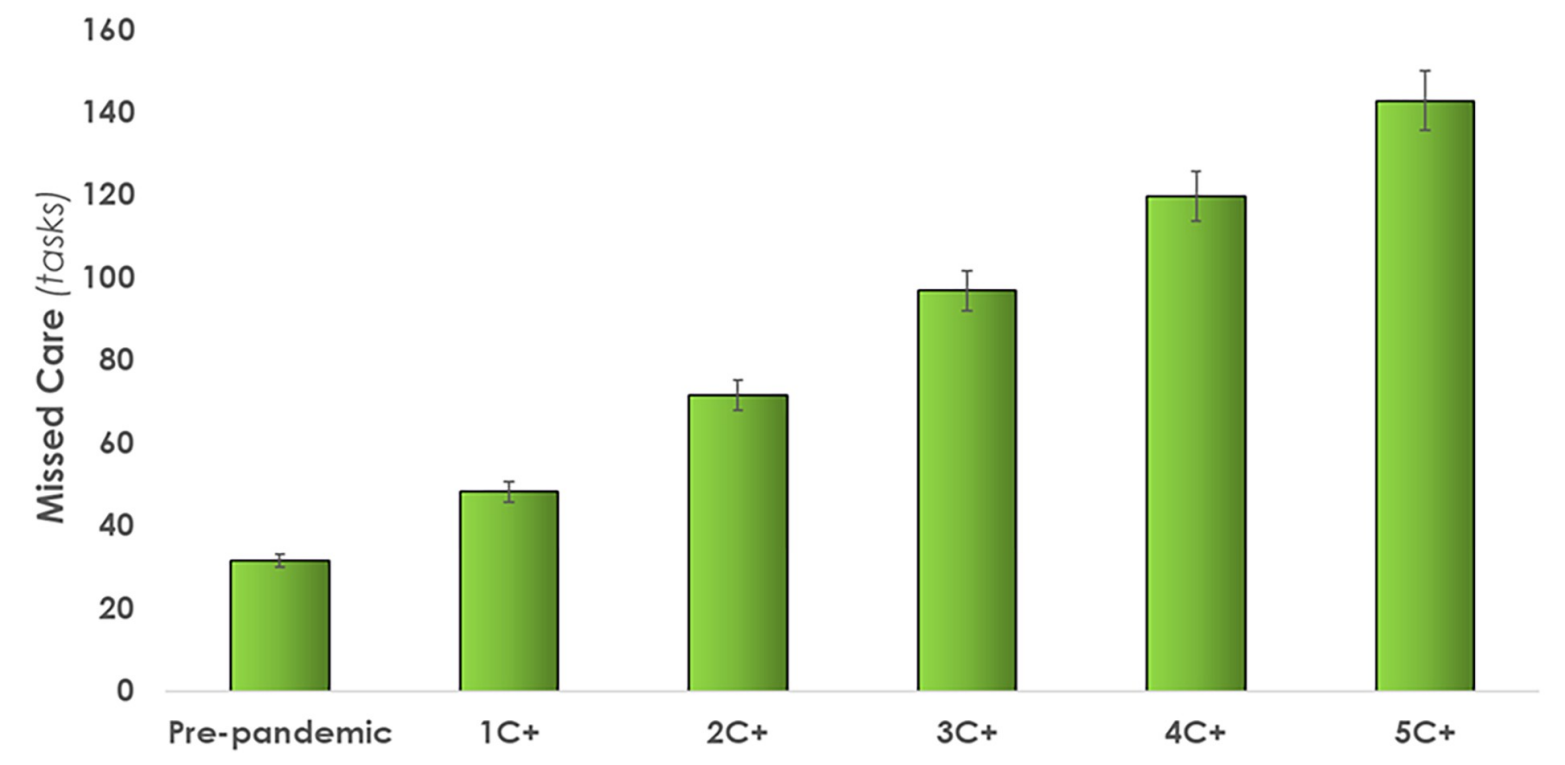
Average number of tasks missed when caring for one to five COVID-19 (C+) positive patients. Error bars indicate standard deviations. Table 3 provides a quantitative overview of the results.

*Missed care*. As shown in Fig 8, a range of 48 (1C+) to 143 (5C+) care tasks are missed when attending to C+ patients, in comparison to 31 missed care tasks pre-pandemic. An increase of up to 353% in the 5C+ condition is observed from the baseline pre-pandemic condition. The highest percentage of missed care tasks were ‘teaching and emotional support’ and ‘documentation’ tasks. In terms of working time, missed care levels would require up to 9.6 hours (5C+ condition) beyond the shift for completion.

**Fig 9:**
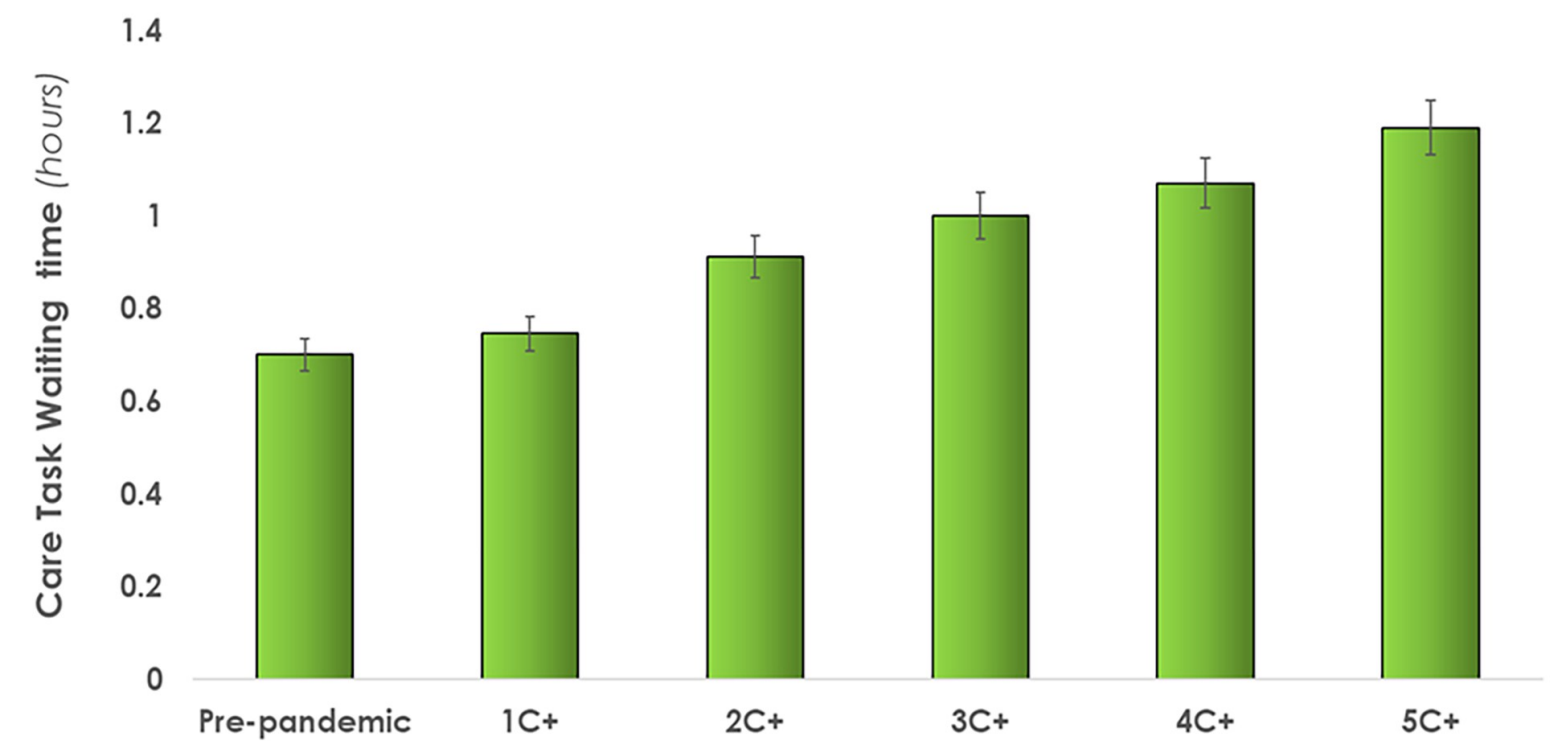
Average care task waiting time within a shift as COVID-19 positive (C+) patients increased from 1 to 5. Error bars indicate standard deviations. Table 3 provides a summary of the quantitative results.

*Care task waiting time*. The average time a care task had to “wait” before the simulant nurse could complete it increased in each condition, up to 70% in the 5C+ condition. As illustrated in Fig 9, care tasks had to wait from 0.75 hours (1 C+) to 1.2 hours (5 C+) when attending to C+ patients. This does not include tasks that were *not* completed at the end of the 12-hour shift.

Table 3 presents a summary of the modelling outcomes categorized by the different experimental conditions. General trends show that as the number of C+ patients increase, the nurse workload increases and the ability to deliver quality of care degrades.

**Table 3:** Summary of the different experimental conditions. Where, C+ = COVID-19 positive patient and the percent change is relative to the Pre-pandemic (baseline) case.

Indicator Category	Indicator Name	Units	Experimental Conditions					
			Pre-pandemic	1 COVID+	2 COVID+	3 COVID+	4 COVID+	5 COVID+
**Nurse Workload**	*Direct Care Time*	*hours*	9.5	8.8 *(-7%)*	7.4 *(-22%)*	5.9 *(-38%)*	4.9 *(-48%)*	3.4 *(-64%)*
	*No*. *of times Donned and Doffed*	*#*	-	31	55	74	91	106
	*Cumulative Donning and Doffing Time*	*hours*	-	2	3.2	4.1	5	6.1
	*Cumulative Distance Walked*	*km*	7.1	8.40 *(17%)*	8.73 *(22%)*	10.37 *(45%)*	10.50 *(46%)*	10.55 *(46%)*
	*Mental Workload*	*tasks*	22	31 *(40%)*	41 *(87%)*	54 *(147%)*	64 *(189%)*	75 *(242%)*
**Quality of Care**	*Missed care*	*tasks*	31.5	48 *(53%)*	72 *(127%)*	97 *(208%)*	120 *(280%)*	143 *(353%)*
	*Task waiting time*	*hours*	0.7	0.75 *(7%)*	0.91 *(30%)*	1.00 *(43%)*	1.07 *(53%)*	1.2 *(70%)*

## 4 Discussion

This study validates an approach to quantifying the workload for nurses, and the related care quality parameters under varying operational conditions, in this case with varying numbers of COVID-19 positive (C+) patients assigned. We do this by adapting the DES model of an acute care medical-surgical unit [14], to reflect a patient population that includes C+ patient care. The medical-surgical unit adapted in this study was upgraded to a COVID-19 unit status, as it was tasked with accommodating the additional influx of C+ patients. The validation study revealed an “excellent” correlation (91%) between real-world and modelled walking patterns as interpreted by Koo and Li [52]. Extensions of this validation, by correlating to measured nurse sensitive health outcomes, patient satisfaction, or nurse occupational health outcomes remains an issue for further research [14, 53–56].

As the results demonstrated in the pre-pandemic condition, a nurse’s workday was already loaded with more tasks that could be completed in a 12-hour shift—a finding consistent with earlier modelling efforts [14]. As nurses were assigned to more C+ patients, they spent less time delivering direct care and more time walking and donning and doffing PPE. In comparison to the baseline pre-pandemic condition, the overall direct care time was reduced by -64% to 3.49 hours when caring for 5C+ patients. This decrease in direct care time (compared to the pre-pandemic condition) can be attributed to certain care tasks taking longer for C+ patients (as illustrated in Table 1). More importantly, the need to perform additional IPAC routines i.e. don and doff PPE [57]. The nurse donned and doffed over 100 times a shift while providing care to 5C+ patients, which equated up to 6.1 hours out of a 12-hour shift. A COVID-19 PPE frequency study by Van Dijk and colleagues [58] revealed an average of eight instances of donning and doffing PPE for one patient in four hours. This finding by Van Dijk and colleagues [58] scales to 24 instances per 12-hour shift, following the trend increase seen here with each additional C+ patient, and with 120 instances for 5C+ patients is in similar range to the 106 instances seen in the simulation.

During the focus groups to discuss how COVID-19 has modified work demands, participants revealed they are now “*rushing care tasks*” and rushing how they wear PPE in order to deliver the same level of care during the pandemic. This rushing increases the risk of infection transmission [59]. This further illustrates how nurses are committed to providing quality patient care even if they put themselves at risk of infection—a phenomenon noted in previous research [60]. Rushing and skipping steps in donning and doffing leads to the healthcare worker getting infected, which in turn results in a “double hit” as the care provider population decreases whilst the infected patient population increases. In addition, if an outbreak occurs in the unit, nurses are forced to quarantine at home, which further puts a strain on the nursing staffing as there is now a reduced pool of nurses employed in the hospital. The unit whose simulation model was created in this study, has already seen two outbreaks. The DES model represents ‘day’ shifts, each consisting of 12-hours with no breaks. Breaks were not programmed into the model because it was observed during the pandemic that nurses were rarely taking breaks. The model can be extended to include breaks. This would further increase workload indicators and deteriorate care quality indicators. As the overworked simulant-nurse (as illustrated from the 12-hour modelling conditions in this study) would now have only 11 hours to perform all care activities.

Model outputs of *distance walked by nurse* showed a plateau effect in which the distance walked had little increase beyond 3 COVID+ patients; less than 0.2 km increase over the 3 conditions This can be interpreted as a plateau effect due to the inability to complete additional care tasks; meaning there was no more time available to attend to additional care tasks and therefore walking for care delivery, and any additional work will simply increase the number of undelivered tasks. This places nurses in the difficult position of having to either rush to complete tasks, with consequent negative implications [59], stay on and work past the end of shift, or engage in triage deciding what tasks can be skipped or passed on to the next shift. In 2016, unpaid and paid overtime for Canadian nurses increased from 19 million hours to 20+ million hours, equivalent to 11,100 full-time equivalent work [61]. Nurses that work beyond a 12-hour shift are more prone to making errors and in some cases making near-death mistakes [7]. This analysis did not adjust nurse-patient ratios, which is one mechanism for workload management. Further model experimentation would be required to identify an optimal mix between the number of patients and infection status for a given unit configuration so as to ensure that all required care is delivered within a working shift.

The DES model reports over 140 tasks being missed, or left undone, at the end of a shift when caring for 5 C+ patients. This is consistent with previously published research where missed care was increased when attending to more acute patients [62]. Of the 143 missed care tasks in the highest workload condition (5C+), most missed care tasks included documentation, teaching and emotional support, and non-patient care, which was consistent with other studies on missed care [62]. This was confirmed qualitatively by the unit nurses who identified documentation, teaching and emotional support and non-patient care tasks as having been most compromised by pandemic-related extra demands. Since C+ patients are not allowed visitors, and thus in-person support from family and friends, the absence of teaching and emotional support from nurses has the potential to negatively impact the mental and emotional wellbeing of patients [63–66]. Nursing professionals are deeply concerned about the health of their patients and not being able to spend time teaching and providing emotional support to all their patients further impacts their mental workload. Unable to keep up with the mental and physical burden, some HCPs have committed suicide [67]. As of January 2021, a total of 26 worldwide HCP COVID-19 related suicide cases have been documented between the ages of 22- to 60-year-olds [68]. Young et al. [69] surveyed 1,685 healthcare workers in the US during the pandemic; nearly half reported severe psychiatric symptoms such as suicidal ideation. The Canadian Union of Public Employees surveyed 2600 nurses on the COVID-19 work demands, where 80% of the respondents reported their workload had "increased a lot" [70]. Our model quantified the associated increase in mental workload as indicated by the dependent variable *tasks in queue*. The average number of tasks waiting to be performed increased by up to 242%, from 22 tasks in pre-pandemic condition to up to 75 care tasks when caring for 5 C+ patients. Given these increased demands, care task waiting time increased up to 1.2 hours, a 70% increase from the baseline condition. The modelling capability developed in this study can be used to examine the mental burden and test strategies to better manage the mental and physical burden.

In simulation model terms, the priority and movement logics for given tasks, and critical inputs to the model, will determine the sequence of execution and, therefore, the nature of tasks that remain incomplete at the end of the simulated shift. By making these priorities and their impacts explicit, this modelling opens the door to revisit and adapt task assignment and priority policies in ways that might minimize the negative effects of the extra workload associated with IPAC routines. Pre-pandemic, it has been reported that some nurses had started to “bundle” care tasks to reduce walking and to streamline care delivery [71]. During the focus groups it was revealed that all nurses have started to do this regularly, especially for C+ patients to reduce donning and doffing time. The element of bundling care tasks was included in this study. While it is possible to configure the model to individual nurse views and care delivery approaches to examine the impacts these differences have, an aim of this research was to explore the impacts of the number of C+ patients on workload. This modelling capability can be extended to explore optimal bundling strategies. Undelivered care can have negative consequences for both patients, causing higher readmission rates [72]. For nurses, having to compromise their intent to deliver high quality care can have negative impacts on both motivation and psychological wellbeing [62]. This is an example of how model outputs can be used to address both patient and nurse outcomes.

### 4.1 Implications to the healthcare systems

The modelling approach demonstrated here integrates available evidence and data to help understand the complex system dynamics including, nurse outcomes (workload) and patient outcomes (quality of care). In this view we model healthcare units as sociotechnical systems [73, 74] in which the system performance, and hence patient care quality, hinges on a system design that does not overload the humans in the system nurse caregivers [75]. The modelling approach demonstrated here can help managers and system decision-makers understand how their system functions under ‘normal’ as well as unexpected circumstances like pandemic scenarios. These models can provide quantitative information on the effects of changes in a wide variety of system parameters, including layout, procedures, policies, and technology application. Models such as the one created here pose potential engines for future decision support tools that can examine a range of decisions affecting the design, delivery and quality of healthcare services. These act as a kind of ‘management flight simulator’ [76, 77], allowing different operational policies (e.g. staffing, patient assignments) and innovation options to be examined for their impact on staff workload and care quality. In this study, the impacts of pandemic-related changes in task sequencing and IPAC routines were measured. Without specific quantitative models of the extra demands of pandemic care routines, healthcare system managers are essentially “flying blind”. The developed modelling approach can help *hospital managers* understand the implications of different scenarios on their staff–and hence on the system as a whole–in a range of possible scenarios. This research has implications for the design of the healthcare system as a whole, including pandemic planning scenarios. Furthermore, this research illuminates and quantifies the workload issues experienced by nurses on the COVID-19 front lines.

Healthcare poses a complex system [78] with many different decision-makers involved in determining the architecture and layout, procedures, policies, and staffing routines. Thus, there is a wide range of stakeholders who might benefit from using such models. These models can help *administrators* understand and predict the impact of work design decisions (e.g. staffing ratios and skill mix) on nurse workload and thereby the quality of care, while *policymakers* can test the consequences of policy tradeoffs and technical design. *Architects* and *technology developers* can examine the workload and care quality implications of different design choices they are considering. Meeting the needs of these stakeholders will require a more routinized approach to model creation which currently requires resource-intensive and extensive in-data collection in order to program.

### 4.2 Limitations and future work

The modelling approach demonstrated here is, by necessity, incomplete. Future work includes adding new model capability to quantify the biomechanical workload and associated musculoskeletal disorder risks faced by staff due to the physical workloads [79]. This can be extended to measures of fatigue [80] which can, in turn, be linked to error making [81] which is a major issue in healthcare systems [82–84]. These indicators can then be calibrated to more distal patient and nurse outcomes such as readmission rates, injury rates, staff turnover, and other workload-sensitive distal outcomes of interest. This study does not provide a “one size fits all” quantitative answer that applies to all COVID care hospital units–rather, it was the development of a modelling approach to proactively test the impact of various C+ and C- patients levels on nurse workload and quality of care. Different acuity levels of each C+ and C- patient categories were not taken into account in this current work, but could be undertaken in the future. The developed modelling capability can be extended to test multiple acuity levels for C+ and C- patients, multiple room spatial distribution options in the ward, single-patient rooms or cohorted wards, and explore different types of bundled care activities. Further, this study did not capture time spent in “student” teaching, a component of the partner hospital. The modelling approach may be extended in the future to quantify the impact of this responsibility. The developed modelling capability can also be extended to test implications of alternative donning and doffing policies (e.g. partial changes). This study used a weighted mode value as an attempt to represent the most experienced nurses in the modelling algorithms. Participants with higher experience were given more weight in calculations. This adaptable modelling approach can be extended to create modelling algorithms specific to individual nurse characteristics or work strategies with regards to movement logic and task performance which could change as a result of the specific experience of the nurse. Similarly, the impacts of different approaches, work routines, and performance times could, in principle, be included to whatever level of detail the model user desires or can afford.

Beyond model capability, there is a need to extend research on the application and use of such tools by key decision-makers in practice. How can such models be deployed in ways that minimize model development time to create useful decision support tools? Further field research is needed to examine the application domain of these models. There is also a need to extend these models into healthcare systems beyond the medical-surgical unit examined here. In this study a medical-surgical unit was selected because the largest proportion of acute care nurses across Canada (24%) work in this area [85]. Long Term Care, Complex Continuing Care, and other clinical care delivery systems can, and should, be examined using these kinds of workload modelling approaches. Process innovation and design of these systems can both be supported using the fundamental modelling approach advocated here.

## 5 Conclusion

This study presents an approach to developing and applying DES to quantify nurse workload and care quality outcomes during COVID-19 pandemic conditions. The model was found to be valid in comparison to actual nurse walking distances with an ICC of 0.91. This study provides quantifiable evidence of the work overload nurses are experiencing during this pandemic and its implications on the quality of care. Increasing the number of C+ patients in a nurse’s assignment, substantially increased nurse workload and decreased the quality of care. Nurses were found donning and doffing PPE up to 106 times a shift, translating into 6.1 hours donning and doffing PPE when caring for 5 C+ patients. This contributed to reducing direct care time down to 3.4 hours. In addition, nurses in this 5 C+ scenario walked up to 10.55km per shift while managing an average of up to 75 care tasks in the task queue. While caring for 5 C+ patients, missed care increased up to 142 tasks, and missed care working time increased up to 9.6 hours, and. care task waiting time increased up to 1.2 hours. This pandemic has pushed an already overworked population of nurses, beyond their limits. 2020 was the 200^th^ birth anniversary of the founder of modern nursing–Florence Nightingale [86]–is this really the legacy of Florence Nightingale?

The modelling approach used here has the potential for use in a wide range of scenario testing. It is sensitive to architecture, care delivery routines and policies, patient characteristics, and even nurse preferences in care delivery approaches. While the model shows promise for decision support, as it integrates the effects of a wide range of decisions normally made by diverse stakeholders, further study is needed to see how this information can be made accessible and useful to these stakeholders to support meaningful improvements in the design and management of healthcare systems under both normal and unexpected operating scenarios such as future pandemics.

## Supporting information

S1 Data (XLSX)
